# Characterisation of a murine model of the late asthmatic response

**DOI:** 10.1186/s12931-017-0541-x

**Published:** 2017-04-11

**Authors:** Katie Baker, Kristof Raemdonck, Robert J. Snelgrove, Maria G. Belvisi, Mark A. Birrell

**Affiliations:** 1 Respiratory Pharmacology, Airway Disease Section, National Heart and Lung Institute, Faculty of Medicine, Imperial College London, Exhibition Road, London, SW7 2AZ UK; 2 Department of Anatomy, Faculty of Medicine, University of Porto, Alameda Prof. Hernâni Monteiro, 4200-319 Porto, Portugal; 3 Center for Health Technology and Services Research (CINTESIS), Faculty of Medicine, University of Porto, Rua Dr. Plácido da Costa, 4200-450 Porto, Portugal; 4 Leukocyte Biology Section, National Heart and Lung Institute, Imperial College London, London, UK; 5 Asthma UK Centre in Allergic Mechanisms of Asthma, Imperial College London, London, UK

## Abstract

**Background:**

The incidence of asthma is increasing at an alarming rate. While the current available therapies are effective, there are associated side effects and they fail to adequately control symptoms in all patient subsets. In the search to understand disease pathogenesis and find effective therapies hypotheses are often tested in animal models before progressing into clinical studies. However, current dogma is that animal model data is often not predictive of clinical outcome. One possible reason for this is the end points measured such as antigen-challenge induced late asthmatic response (LAR) is often used in early clinical development, but seldom in animal model systems. As the mouse is typically selected as preferred species for pre-clinical models, we wanted to characterise and probe the validity of a murine model exhibiting an allergen induced LAR.

**Methods:**

C57BL/6 mice were sensitised with antigen and subsequently topically challenged with the same antigen. The role of Alum^TM^ adjuvant, glucocorticoid, long acting muscarinic receptor antagonist (LAMA), TRPA1, CD4^+^ and CD8^+^ T cells, B cells, Mast cells and IgE were determined in the LAR using genetically modified mice and a range of pharmacological tools.

**Results:**

Our data showed that unlike other features of asthma (e.g. cellular inflammation, elevated IgE levels and airway hyper-reactivity (AHR) the LAR required Alum^TM^adjuvant. Furthermore, the LAR appeared to be sensitive to glucocorticoid and required CD4^+^ T cells. Unlike in other species studied, the LAR was not sensitive to LAMA treatment nor required the TRPA1 ion channel, suggesting that airway sensory nerves are not involved in the LAR in this species. Furthermore, the data suggested that CD8^+^ T cells and the mast cell—B-cell - IgE axis appear to be protective in this murine model.

**Conclusion:**

Together we can conclude that this model does feature steroid sensitive, CD4^+^ T cell dependent, allergen induced LAR. However, collectively our data questions the validity of using the murine pre-clinical model of LAR in the assessment of future asthma therapies.

**Electronic supplementary material:**

The online version of this article (doi:10.1186/s12931-017-0541-x) contains supplementary material, which is available to authorized users.

## Background

Asthma is defined as a heterogeneous disease, usually characterized by chronic airway inflammation. Patients generally present with a history of respiratory symptoms such as wheeze, shortness of breath, chest tightness and cough that vary over time and in intensity, together with variable expiratory airflow limitation [1–4]. It affects approximately 300 million people worldwide and the World Health Organisation estimates asthma to represent 1% of the total global disease burden [5, 6]. The prevalence of asthma is still on the rise and escalating healthcare costs are making it a global health concern [3, 5–8] Asthma is characterised by airway hyperresponsiveness (AHR), reversible bronchoconstriction, airway remodelling events and chronic inflammation. A wide range of environmental and endogenous stimuli have been shown to trigger asthma symptoms including; allergens, exercise and cold air [9, 10]. Recent attempts at phenotyping asthma have adopted cluster based approaches whereby patients are grouped according to a multitude of possible features such as age of onset, atopic status, severity of airways obstruction and medication requirements. Atopic asthma is associated with allergy (elevated levels of allergen specific IgE) and atopic sensitisation has been implied as an important determinant in the development of asthma.

Allergen inhalation challenge in patients with mild asthma is considered to be a useful model for understanding the mechanisms involved in the pathophysiology of asthma and also to be predictive of therapeutic utility. Allergen inhalation challenge results in a biphasic bronchoconstrictor response, an early asthmatic response (EAR) and a late asthmatic response (LAR) [11, 12]. The EAR usually takes place within 1 h following aeroallergen exposure and is associated with the allergen causing cross-linking of the IgE on mast cells leading to mast cell degranulation and the release of inflammatory mediators such as histamine and cysteinyl-leukotrienes [13–15]. The Late Asthmatic Response (LAR) refers to a more prolonged bronchoconstriction event taking place approximately 3–8 h following contact with aeroallergen [11, 12], however, the aetiology behind the response is less well understood than that of the EAR. Studies so far exploring this particular feature of asthma have concluded that it is likely that the LAR is at least in part driven by inflammation. This conclusion is largely based on the fact that the response is accompanied by cellular inflammatory influx into the lung along with the demonstration that steroid treatment impacts on the response [16, 17]. Others have suggested alternative mechanisms behind the LAR such as a possible role for airway sensory nerves [18].

Pre-clinical animal models are used in the drug discovery process to understand disease pathogenesis and to test the utility of potential therapeutics [12]. The mouse is being increasingly utilised in pre-clinical asthma models due to the availability of new genetically modified strains [19–21]. However, it is increasingly being reported that data obtained from animal models is not predictive of clinical therapeutic outcome [22, 23]. One possible reason for this could be that the murine models most frequently used exhibit a number of asthmatic endpoints such as AHR, airway inflammation and airway remodelling events; but do not always demonstrate the same endpoints as those used in early clinical development such as allergen induced LAR. Very few publications have described a murine LAR and the nature of the response has not previously been investigated. Therefore, the purpose of this investigation was to characterise an OVA-driven mouse model of the LAR including probing the role of sensory nerves and key allergic effector cells, and the requirement for adjuvant.

## Methods

### Animals

All experimental protocols were approved by a local ethical review process and strictly adhered to the Animals (Scientific Procedures) Act 1986 UK Home Office guidelines and performed according to the ARRIVE guidelines. All animals were housed in individually ventilated cages (IVC) and a 12-h light-dark cycle maintained. Prior to and during experimental periods, food and water was supplied *ad libitum*. C57BL/6 wildtype (Harlan, UK), mast-cell knockout [KitW-sh] (Jackson Laboratories Ltd, USA), B-cell knockout (Swiss Immunological Mouse Repository), *Trpa1* knockout (Jackson Laboratories Ltd, USA and Prof. Peter Zygmunt, Sweden - originally developed by Prof David Julius USA), CD4 knockout (Swiss Immunological Mouse Repository), CD8 knockout (Swiss Immunological Mouse Repository) and IgE knockout (Swiss Immunological Mouse Repository) male mice aged 8–12 weeks were bred in house. In the experiments detailed below a randomization procedure used to allocate the animals to various groups.

### The OVA driven mouse model of LAR

Male mice (20–25 g) were sensitised with OVA in Alum^TM^ (50 μg per mouse, 500 μl, i.p.) or vehicle (Alum^TM^ diluted 1:1 with saline, 500 μl, i.p) on days 0 and 14 in their home cage. On day 28, mice were given a single intra-tracheal (i.t.) dose of OVA (2% OVA, 25 μl) as described [18, 24]. For the i.t. dosing, mice were placed in a Perspex chamber connected to an anaesthetic machine (Bowring Medical Engineering Ltd, Witney, UK) and exposed to 4% isoflurane in 0.5% oxygen until sufficiently anaesthetised. Mice were dosed into the trachea using a dosing gavage and then monitored until fully recovered. Immediately following recovery from i.t. dosing, mice were placed in WBP chambers (Buxco Electronics, USA) and pressure changes continuously recorded by BuxcoFinepointe Software (Buxco Electronics, USA). The average Penh was recorded at 10 min intervals for a total duration of 12 h and was used for analysis.

In order to assess the importance of Alum^TM^adjuvant within this model, the protocol was adapted by sensitizing different groups with and without Alum^TM^adjuvant. The effect of a clinically used glucocorticosteroid, Budesonide (given at an effective supra-maximal dose of 3 mg/kg at 10 ml/kg, p.o., [18, 25–29]) or vehicle (0.5% methylcellulose, 0.2% Tween80 in water) dosed 60 min before allergen challenge, was assessed. The effect of LAMAs was tested by dosing mice with Tiotropium (0.001 mg/kg i.t.), Glycopyrrolate (0.01 mg/kg i.t.) or vehicle (0.5% ethanol in saline, 50 μl i.n.) one hour before allergen challenge. Effective doses were chosen based on dose-response experiments in OVA driven mouse models of AHR (see next section for methodology). Immediately following recovery from allergen challenge, mice were placed in WBP chambers and Penh recorded for 12 h as previously detailed [18, 30]. The role of TRPA1, CD4^+^ cells, CD8^+^ cells, mast cells, B-cells and IgE was investigated by applying the relevant knockout mice to the model and comparing responses to that of wildtype mice.

### The OVA driven mouse model of AHR

Mice were dosed 50 ul i.n. with tiotropium (1 μg-0.1 mg/kg), glycopryrrolate (0.01–10 mg/kg) or vehicle (saline, i.n.) 240 min before challenge. Mice were then placed in the Penh chambers and allowed to acclimatise for 2 min followed by a 2-min recording of the baseline. They were then exposed to saline and methacholine (2–32 mg/ml, every 10 mins). Whole body plethysmography (WBP; Penh) was used to assess changes in airway tone. Penh was recorded for 10 min between doses. Animals were culled following the experiment.

### Measurement of plasma IgE

Animals were euthanised via overdose with Sodium Pentobarbitone (200 mg/kg i.p.). Blood was attained via cardiac puncture with a heparinised syringe. Samples were then centrifuged (1398 g, 10 min, 4 °C) and the plasma supernatant transferred to eppendorfs and stored at −20 °C until subsequent analysis. The level of plasma IgE was used as a marker of allergic sensitisation and measured using Enzyme Linked Immunosorbant Assay (ELISA). Total and allergen specific IgE levels were measured using BD OptEIATM set for mouse immunoglobulin E (BD Biosciences, Oxford, UK) in accordance with the manufacturer’s instructions (see Additional file 1).

### Bronchoalveolar lavage fluid (BALF)

The trachea was exposed via blunt dissection and cannulated. A syringe was used to introduce RPMI media (Life Technologies) into the lungs for 30 s, before being withdrawn. 0.3 mls was introduced 3 times. The samples were then pooled to give 1 sample per animal, before being prepared for total and differential cell counts. The total leukocyte cell counts in BAL fluid were attained using a Sysmex XP-300 automated cell counter (Sysmex Ltd., UK). Slides for differential cell counts were prepared using 100 μl BAL fluid in a cytospin (807 g, 5 min, room temperature, low acceleration) (Shandon, Runcorn, UK). Slides were stained using a Hema-tek 2000 automated slide stainer (Ames Co., Elkhart, USA) using ACCUSTAIN® modified Wright-Giemsa stain (Sigma). Slides were analysed under light microscopy at ×40 magnification by an observer blinded to the specimen identities. Differential counts were completed on 200 cells per slide using standard morphological criteria. The percentage of macrophages/monocytes, neutrophils, eosinophils and lymphocytes were calculated. Macrophages and monocytes were counted as one group. Other cell types such as epithelial cells and red blood cells were ignored.

### Flow cytometry

Flow cytometry was used to validate the lung tissue cell populations within the genetically modified and wildtype mice. Naïve animals were culled via overdose with Sodium Pentobarbitone (200 mg/kg i.p.). The systemic circulation was then perfused. The lungs were removed, weighed and finely chopped using a Mcllwain tissue chopper (Campden Instruments Ltd, UK) and transferred to 1 ml Roswell Park Memorial Institute media (RPMI)/Penicillin-Streptomycin. This was then added to 4mls RPMI supplemented with 10% Foetal Bovine Serum (FBS), collagenase (1 mg/ml) and DNase (25 μg/ml) and incubated in a water bath with gentle agitation for 1 h at 37 °C. Following this, samples were filtered using a 70 μm mesh sieve. The samples then underwent two rounds of centrifugation at 807 g, discarding the supernatant and re-suspending the pellet in 10 mls RPMI (supplemented as previous) each time. For each sample, a total leukocyte cell count was performed using a Sysmex XP-300 cell counter (Sysmex Ltd., UK). Cells were stained for surface markers as detailed in Snelgrove et al., 2008 [31]. Cell types were characterised by their forward and side scatter profiles and by their specific phenotypes (See Table 1). Unstained controls were used to account for the auto-fluorescence of the samples and the Photomultiplier tube (PMT) gains set accordingly. Single stained controls were used to account for spectral overlap and the flow cytometer compensations set accordingly. Gates to assess positive and negative staining were set using standard fluorescence minus one (FMO) controls for each monoclonal antibody conjugate.

**Table 1:** Outlining surface marker antigens and relevant conjugated monoclonal antibodies used to identify various cell populations

Cell Type	Surface marker Phenotype	Monoclonal Antibody Conjugate
B-cells	CD19^+^	Rat anti-mouse CD19 (FITC)
CD4^+^ T-cells	CD4^+^	CD4 (PerCP)
CD8^+^ T-cells	CD8^+^	CD8 (APC)
Neutrophils	Ly-6G^high^ CD11b^high^ CD11c^low^ F4/80^low^	Ly6G (FITC) CD11b (PerCP) CD11c (APC) F4/80 (PE)
Eosinophils	CD11b^high^ CD11c^low^ Ly6G^low^ SiglecF^high^	CD11b (PerCP) CD11c (APC) Ly6G (FITC) SiglecF (PE)
Alveolar Macrophages	CD11b^low-intermediate^ CD11c^high^ F4/80^high^ Ly-6G^low^	CD11b (PerCP) CD11c (APC) F4/80 (PE) Ly6G (FITC)
Inflammatory monocytes/tissue macrophages	CD11b^high^ CD11c^low^ F4/80^high^	CD11b (PerCP) CD11c (APC) F4/80 (PE)

### Histology

Histology was used to validate the lung tissue mast cell populations within the genetically modified and wildtype mice. Mast cells were identified using a standard Toluidine blue histological stain. Mice were culled via overdose with sodium Pentobarbitone and the systemic circulation perfused as described in the previous section. The trachea was then cannulated and the lungs superfused with formalin before being placed in formalin for 24 h. Following this, they were transferred into 70% ethanol until paraffin wax embedding and slicing could take place. The paraffin embedded lung samples were cut into 4 μm sections. The sections were stained using a standard Toluidine Blue and eosin counterstain protocol as in Yagil et al. (2012) [32]. Briefly, the lung sections were dewaxed using Histochoice clearing agent (Sigma, UK) and rehydrated in a series of ethanol dilutions (100, 90, 70%). The slices were then washed in deionised water and stained in 0.1% Toluidine Blue (Sigma, UK) for 5 min. Sections were then washed in distilled water before counterstaining with Eosin-Phloxine solution (Sigma, UK) for 1 min. A final wash in distilled water then took place before the slices were dehydrated using a series of ethanol dilutions (70, 90, 100%). The slices were left to dry at room temperature and mounted onto glass slides [32]. The stained sections were analysed under light microscopy at ×40 magnification, the observer blinded to the specimen identities. The numbers of mast cells per slide (3 slides per lung sample) were counted.

### Compounds and materials

Isoflurane was from Abbott Laboratories (UK). BD OptEIATM set for mouse immunoglobulin E was from BD Biosciences (US). Medical Oxygen was from BOC Industrial Gases (UK). FBS and Fixable near-IR dead cell stain kit for 633/635 nm excitation was from Invitrogen (UK). Collagenase and DNase was from Roche Diagnostics (Germany). Alum^TM^ was from ThermoFisher Scientific (UK). Glycopyrrolate, ethanol and 2 N H_2_SO_4_ was from VWR (UK). Tiotropium was a gift from Boehringer Ingelheim (Germany). Sterile saline was purchased from Fresenius Kabi Limited (UK) and pentobarbitone from National Veterinary Services Limited (UK). All other agents were purchased from Sigma-Aldrich (UK) unless otherwise described.

### Statistics

Data was expressed as mean ± S.E.M of n observations. A *p* value < 0.05 was taken as statistically significant, the actual test used is indicated in the figure legends.

## Results

### Validation of the LAR model using a clinically relevant glucocorticosteroid

**Fig. 1:**
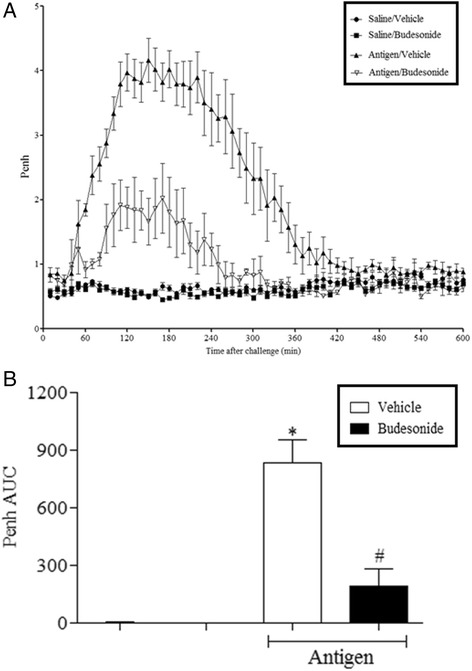
The effect of Budesonide treatment in the mouse OVA-driven LAR. Immediately following recovery from allergen challenge, mice were placed in WBP chambers and Penh recorded for 12 h. The *two bars* on the left are the data from the saline challenge/vehicle treated and saline challenged/drug treated, control groups. Data is expressed as **a**) Penh average over the recording time period; **b**) Penh Area Under Curve. Data expressed as mean ± s.e.m. *n* = 5–8. Mann-Whitney *U*-test. *p < 0.05 Vehicle/Saline compared to Vehicle/Ovalbumin. #*p* < 0.05 Budesonide (3 mg/kg)/Ovalbumin compared to Vehicle/Ovalbumin

The effect of high dose budesonide was assessed In order to evaluate the clinical relevance of the OVA driven mouse LAR model. OVA challenge resulted in a marked LAR in OVA sensitised mice. This response peaked at 3 h and lasted for up to 8 h after challenge. The saline sensitised mice did not display a response following OVA challenge. Budesonide treatment resulted in a significantly decreased LAR in the OVA sensitised/OVA challenged mice when compared with vehicle treated mice (Fig. 1). No effect of budesonide was observed on the Penh baseline recordings.

### Assessing the importance of Alum^TM^ adjuvant in murine asthma models

**Fig. 2:**
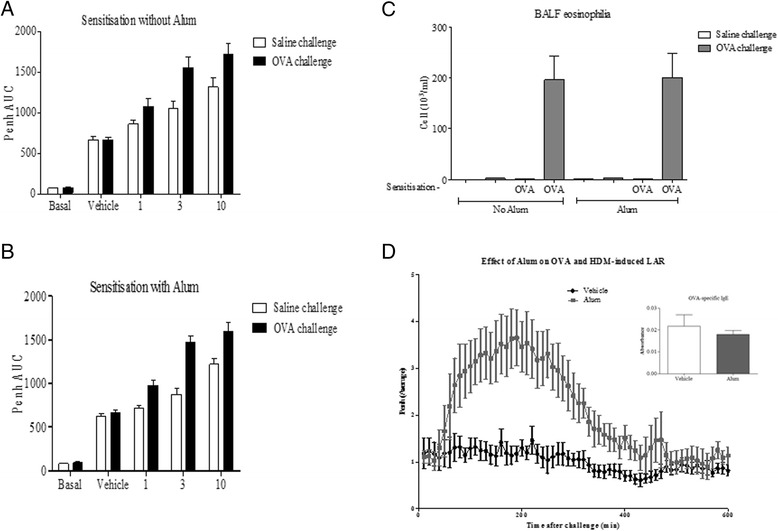
Assessing the importance of Alum in murine asthma models. Mice underwent antigen sensitisation either **a**) without Alum^TM^ or **b**) with Alum^TM^ before undergoing increasing Methacholine challenge and Penh recording at 10 min intervals. Following this **c**) BALF eosinophilia was assessed. **d**) Mice also underwent antigen sensitisation with and without Alum^TM^ before OVA induced LAR was recorded. Data expressed as mean ± s.e.m. *n* = 8

Traditionally, adjuvant has been reported as a requirement when utilising OVA to model features of allergic asthma within murine models. However, when investigating the requirement for Alum^TM^ adjuvant within the AHR model, it was found not to be essential. Indeed, the group sensitised with vehicle plus OVA conferred comparable AHR to the group sensitised with Alum^TM^ plus OVA (see Fig. 2 a, b). Eosinophilia was also comparable between the groups (Fig. 2c). Interestingly, Alum^TM^ adjuvant was found to be a requirement in the sensitisation process for mice to elicit an LAR. Mice sensitised with Alum^TM^ in addition to OVA antigen elicited a distinct LAR, while mice sensitised with vehicle in addition to OVA antigen did not demonstrate a response (Fig. 2d). It should be noted that negative controls were also included in investigations (i.e. mice sensitised with Alum^TM^ plus saline without antigen and allergen challenged) and did not elicit responses (data not shown).

### Exploring the role of airway sensory nerves in the murine LAR model

**Fig. 3:**
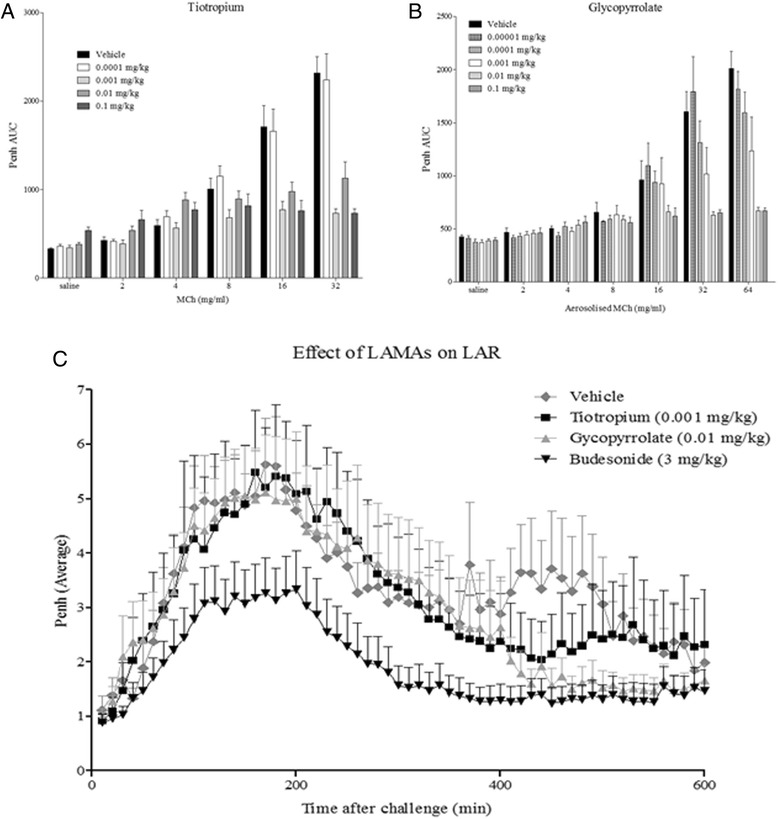
The Role of Airway Sensory Nerves in the mouse OVA-driven LAR model. Mice underwent a dose response to inhaled Methacholine having previously been dosed with **a**) Tiotropium or **b**) Glycopyrrolate in order to establish an effective dose for the LAR investigation. Following this **c**) Tiotropium, Glycopyrrolate and Budesonide were applied to the OVA driven mouse model of LAR. Data expressed as mean ± s.e.m. *n* = 8

**Fig. 4:**
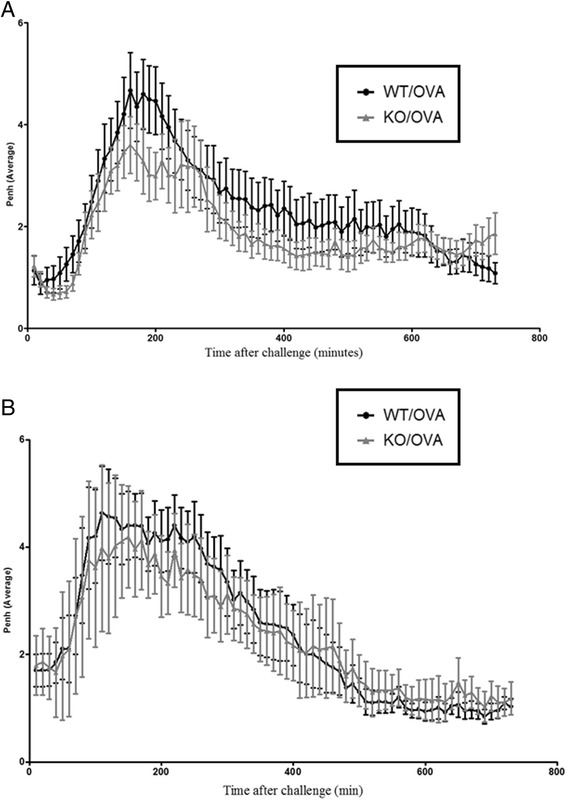
Assessing the importance of TRPA1 in the mouse OVA-driven LAR model. TRPA1−/− mice were obtained from either **a**) Jackson laboratories or **b**) David Julius Laboratory before being applied to the mouse OVA-driven LAR model. Data expressed as mean ± s.e.m. *n* = 8

Glycopyrrolate (over the range of MCh challenge doses the approximate ED_50_ was between 0.001 and 0.01 mg/kg, with the mean Emax of 0.01 mg/kg) and Tiotropium (the approximate ED_50_ was between 0.0001 and 0.001 mg/kg, with the mean Emax of 0.001 mg/kg) significantly attenuated AHR to methacholine. The Emax dose values were chosen to use in the mouse LAR model. Glycopyrrolate and Tiotropium failed to impact upon the LAR response but the positive control (Budesonide) significantly attenuated the LAR (Fig. 3). Interestingly, the LAR was still evident in TRPA1^−/−^ mice when compared with wildtype controls. In order to confirm the data obtained in the TRPA1^−/−^ mice obtained from Jackson Laboratories an alternative colony of TRPA1−/− mice originally developed by the Julius Lab were obtained and utilised. However, similar results were obtained consistent with the previous experiments showing no difference in the LAR response between the TRPA1^−/−^ and wildtype mice (Fig. 4).

### Investigating the role of key allergic effector cells in the OVA driven mouse model of LAR

**Fig. 5:**
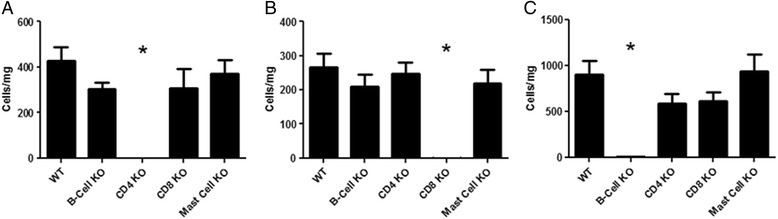
Validation of Lymphocyte Populations in Genetically Modified Mice by Flow Cytometry. Leukocytes from the lung tissue of wildtype (C57BL/6), B-cell−/−, CD4−/−, CD8−/− and Mast-cell−/− naïve male mice were attained via enzymatic digest. Flow Cytometry was used to assess the numbers of: **a**) CD4+ cells; **b**) CD8+ cells and **c**) CD19+ cells. Data is expressed as mean cell number per mg of lung tissue ± s.e.m. *n* = 6–8. One-way ANOVA, Kruskal-Wallis test with post-hoc comparisons using Dunn’s multiple comparison test. *p < 0.05 compared to wildtype control group

**Fig. 6:**
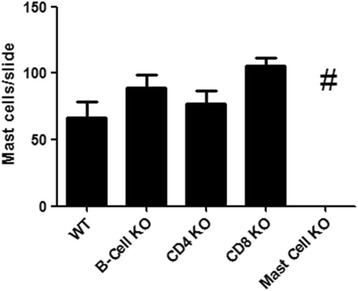
Validation of Mast Cell Populations in Genetically Modified Mice by Histology. Lung tissue slices were obtained from wildtype C57BL/6, B-cell^−/−^, CD4^−/−^, CD8^−/−^ and Mast-cell^−/−^ naïve male mice. They were stained utilising a standard Toluidine Blue histological stain and numbers of mast cells per slide were counted (×40 magnification) by a blinded observer. Data is expressed as mean cell number per slide ± s.e.m. *n* = 6–8. One-way ANOVA, Kruskall-Wallis test with post-hoc comparisons using Dunn’s multiple comparison test. #*p* < 0.05 compared to wildtype control group

Prior to investigating a role for various key allergic effector cells in the LAR response we evaluated the phenotype of the CD4^−/−^, CD8^−/−^, B-cell^−/−^ and mast-cell^−/−^ mice using flow cytometry and histology. The CD4^−/−^, CD8^−/−^ and B-cell^−/−^ GM mice were all confirmed to be deficient of their specific knockout cell via flow cytometry (See Fig. 5). Lung tissue populations of CD4^+^, CD8^+^ and CD19^+^ cells in the knockout mice were found to be comparable to those seen in wildtype mice where the cell type was not the deficient population (data not shown). The mast cell knockout mice were confirmed to be deficient of lung mast cells using the standard histological stain Toluidine Blue on lung tissue slices. No significant differences with respect to the mast cell population in the other GM strains were found compared with the wildtype group (Fig. 6). Baseline levels of eosinophils, neutrophils, alveolar macrophages and monocytes/tissue macrophages were assessed by flow cytometry in wildtype mice compared with the various transgenic mice. No significant differences in these cell types were found across the GM strains compared with the wildtype group (data not shown).

**Fig. 7:**
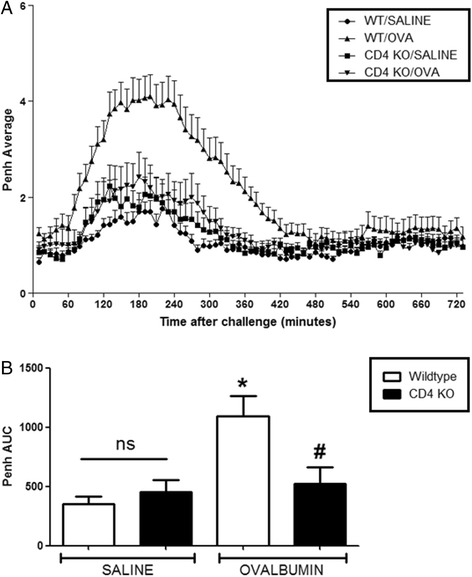
The Role of CD4^+^ Cells in the OVA-driven Mouse Model of LAR. Immediately following recovery from allergen challenge, mice were placed in WBP chambers and Penh recorded for 12 h. Data is expressed as **a**) Penh average over the recording time period; **b**) Penh Area *Under Curve*. Data expressed as mean ± s.e.m. *n* = 13–21. Mann-Whitney *U*-test. *p < 0.05 wildtype *Saline* sensitised compared to wildtype *Ovalbumin* sensitised groups. #*p* < 0.05 CD4^−/−^ *Ovalbumin* sensitised compared to wildtype *Ovalbumin* sensitised groups. No significant difference between groups is denoted *ns*

**Fig. 8:**
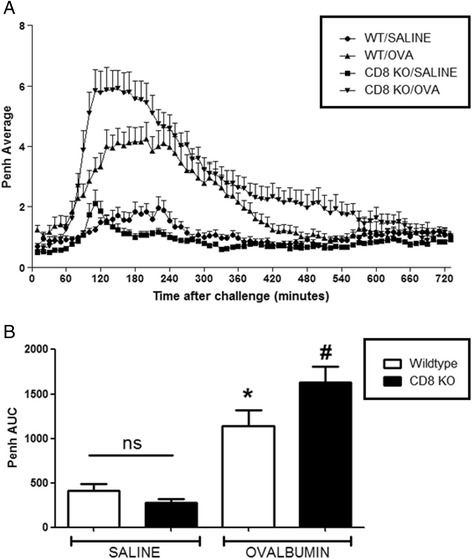
The Role of CD8^+^ Cells in the OVA-driven Mouse Model of LAR. Immediately following recovery from allergen challenge, mice were placed in WBP chambers and Penh recorded for 12 h. Data is expressed as **a**) Penh average over the recording time period; **b**) Penh Area *Under Curve*. Data expressed as mean ± s.e.m. *n* = 14–17. Mann-Whitney *U*-test. *p < 0.05 wildtype *Saline* sensitised compared to wildtype *Ovalbumin* sensitised groups. #*p* < 0.05 CD8^−/−^ Ovalbumin sensitised compared to wildtype *Ovalbumin* sensitised groups. No significant difference between groups is denoted *ns*

**Fig. 9:**
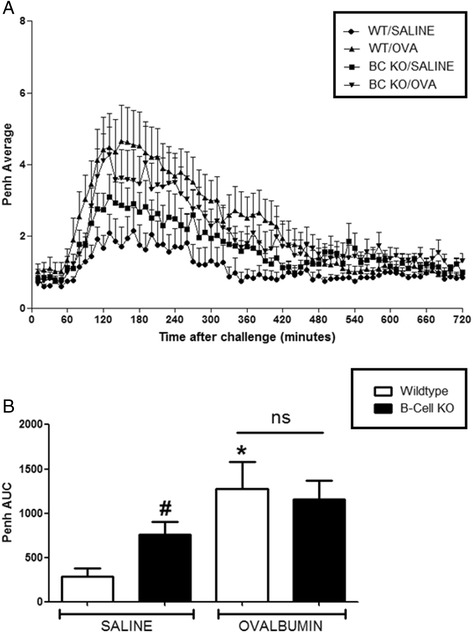
The Role of B-Cells in the OVA-driven Mouse Model of LAR. Immediately following recovery from allergen challenge, mice were placed in WBP chambers and Penh recorded for 12 h. Data is expressed as **a**) Penh average over the recording time period; **b**) Penh Area *Under Curve*. Data expressed as mean ± s.e.m. *n* = 12–14. Mann-Whitney *U*-test. *p < 0.05 wildtype *Saline* sensitised compared to wildtype *Ovalbumin* sensitised groups. #*p* < 0.05 wildtype *Saline* sensitised compared to B-cell^−/−^Saline sensitised groups. No significant difference between groups is denoted *ns*

**Fig. 10:**
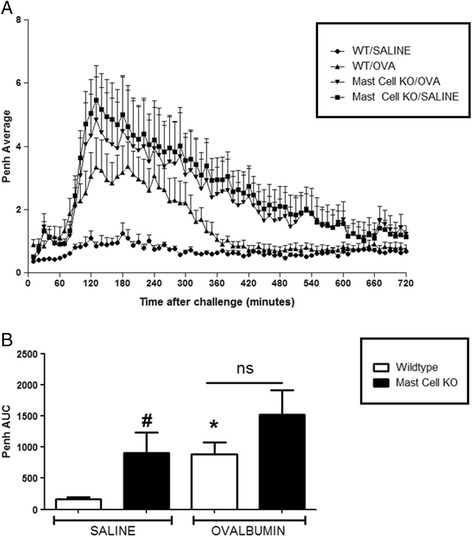
The Role of Mast Cells in the OVA-driven Mouse Model of LAR. Immediately following recovery from allergen challenge, mice were placed in WBP chambers and Penh recorded for 12 h. Data is expressed as **a**) Penh average over the recording time period; **b**) Penh Area *Under Curve*. Data expressed as mean ± s.e.m. *n* = 8–12. Mann-Whitney *U*-test. *p < 0.05 wildtype *Saline* sensitised compared to wildtype *Ovalbumin* sensitised groups. #*p* < 0.05 wildtype *Saline* sensitised compared to Mast cell^−/−^Saline sensitised groups. No significant difference between groups is denoted *ns*

**Fig. 11:**
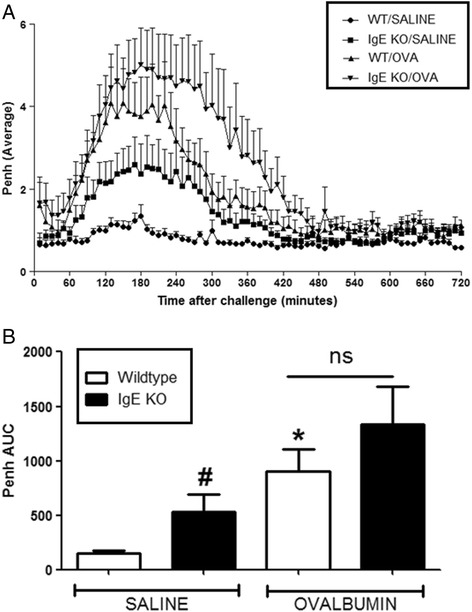
The Role of IgE in the OVA-driven Mouse Model of LAR. Immediately following recovery from allergen challenge, mice were placed in WBP chambers and Penh recorded for 12 h. Data is expressed as **a**) Penh average over the recording time period and **b**) Penh Area *Under Curve*. Data expressed as mean ± s.e.m. *n* = 6–9. Mann-Whitney *U*-test. *p < 0.05 wildtype *Saline* sensitised compared to wildtype *Ovalbumin* sensitised groups. #*p* < 0.05 wildtype Saline sensitised compared to IgE^−/−^Saline sensitised groups. No significant difference between groups is denoted *ns*

**Fig. 12:**
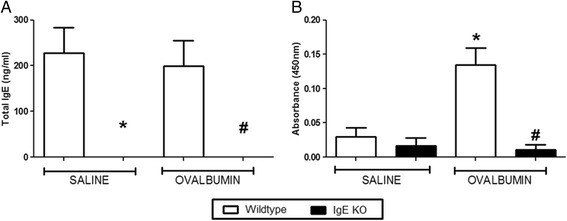
Plasma IgE Levels in IgE Deficient Mice. Following Penh recordings, blood samples were attained via cardiac puncture and plasma samples assessed for **a**) Total IgE and **b**) OVA-Specific IgE via ELISA. IgE knockout mice in comparison to wildtype groups were assessed. Data is expressed as raw absorbance values taken at 450 nm ± s.e.m. *n* = 6–9. Mann-Whitney *U*-test. *p < 0.05 wildtype Saline sensitised compared to Wildtype Ovalbumin sensitised groups. #*p* < 0.05 IgE^−/−^ Ovalbumin sensitised compared to wildtype Ovalbumin sensitised groups

**Fig. 13:**
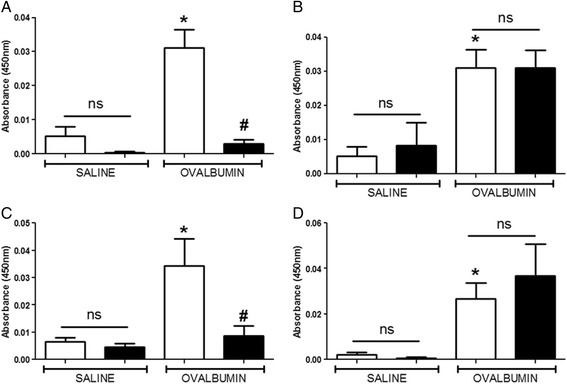
Plasma OVA-Specific IgE Levels in Cell Deficient Mice. Following Penh recordings, blood samples were attained via cardiac puncture and plasma samples assessed for OVA-Specific IgE via ELISA. Genetically modified strains in comparison to wildtype groups assessed were: **a**) CD4^−/−^; **b**) CD8^−/−^; **c**) B-cell^−/−^ and **d**) Mast cell^−/−^. Data is expressed as raw absorbance values taken at 450 nm ± s.e.m. *n* = 6–11. Mann-Whitney *U*-test. *p < 0.05 wildtype Saline sensitised compared to wildtype Ovalbumin sensitised groups. #*p* < 0.05 Knockout Ovalbumin sensitised compared to wildtype Ovalbumin sensitised groups. No significant difference between groups is denoted *ns*

For all experiments, OVA challenge resulted in a marked LAR in wildtype OVA sensitised mice which peaked at 3 h and lasted for up to 8 h after challenge, compared to saline sensitised wildtype mice. CD4^−/−^ and CD8^−/−^ mice exhibited a baseline response comparable to saline sensitised wildtype mice following OVA challenge. A significant attenuation of response was observed in the OVA sensitized CD4^−/−^ mice compared to OVA sensitised wildtype group (see Fig. 7). Interestingly, CD8^−/−^ OVA sensitised and challenged mice exhibited a significantly enhanced response compared to the wildtype OVA sensitised and challenged group (see Fig. 8). Surprisingly, the saline sensitised mast cell^−/−^, B-cell^−/−^ and IgE^−/−^ mice had a significantly enhanced response to OVA challenge compared to the saline sensitised wildtype mice. Even more surprisingly, the OVA-sensitised mast cell^−/−^, B-cell^−/−^ and IgE^−/−^groups displayed responses to OVA challenge which were not significantly different to that seen in the OVA sensitised wildtype group (Figs. 9, 10 and 11). As expected the total and antigen specific IgE plasma levels were abolished in the IgE^−/−^ mice (Fig. 12). The levels of antigen specific IgE levels were also significantly attenuated in the CD4^−/−^ and B cell^−/−^ mice, but not the CD8^−/−^ or mast cell knockout mice (Fig. 13).

## Discussion

In the search for effective new therapeutics for asthma patient’s preclinical models are often utilised to test hypotheses before progression into clinical studies. However, current dogma suggests that preclinical model systems, particularly in the mouse, are often not predictive of therapeutic outcome. Therefore, the aim of this study was to investigate the utility of a mouse model of the LAR as a pre-clinical model, predictive of clinical allergen challenge studies, upon which to assess future asthma therapies. Validation of the model was judged by assessing the impact of a clinically effective glucocorticosteroid, long acting muscarinic receptor and TRPA1 antagonists (previously shown to be effective in the rat LAR response) the need for an adjuvant (Alum^TM^), and a role for key allergic effector cells within the LAR model. Throughout these investigations, Penh was used as an arbitrary measure of airway constriction in order to assess lung function within the models. Although some controversy still exists surrounding the use of Penh, previous work by Raemdonck et al. (2012) [18] described a role for neuronal reflexes in the LAR and so a conscious modeling system was required. There are currently no alternative ways of attaining these measurements without using restraint, and licensing restrictions would not allow the use of restraint for the prolonged periods of time necessary to achieve the LAR response.

In asthmatic patients, the LAR typically occurs 3–8 h following allergen exposure [11]. This model demonstrated response time points similar to these with a peak response at 3 h and a return to baseline at 8 h post challenge. Budesonide was successful in significantly attenuating the responses in the model, suggesting that the model was displaying bronchoconstriction consistent with the LAR, not the EAR, consistent with clinical studies. In the AHR model, Alum^TM^ adjuvant was found to be dispensable within the sensitisation process for mice to elicit a response. This was also the case when observing eosinophilia within the models. In contrast, in the murine LAR model, Alum^TM^ was found to be a requirement in the sensitisation process for mice to display an LAR. This suggests that different mechanisms may be driving the AHR and LAR. Numerous theories for the mechanism of action of Alum^TM^ have been suggested but overall the mechanisms driving the adjuvant activity of Alum^TM^ and its contribution to the LAR remain a mystery but could question the clinical relevance of the LAR in this model.

The aetiology of the LAR has been associated with sensory nerve interactions. Raemdonck et al. (2012) showed that the LAR but not the EAR was lost under general anaesthesia in the Brown Norway rat. Further investigation led to the conclusion that stimulation of airway sensory nerves via TRPA1 channels leading to parasympathetic, cholinergic constrictor responses, is involved in driving the allergen induced LAR. Glycopyrrolate and Tiotropium, used at doses that blocked bronchoconstriction to methacholine in the AHR model, failed to impact upon the response within the OVA driven murine model of LAR. This suggests that airway sensory nerves are not involved in the mechanisms driving the LAR in the mouse model. This is in contrast to previous findings in the more established Brown Norway rat model of LAR, but also in a Guinea-pig model of LAR [11, 33]. When two separate colonies of TRPA1 knockout mice were utilized there was still no attenuation in the LAR response when compared with wildtype controls. This suggests that the TRPA1 channel is not essential in the mouse model of LAR and argues against the contribution of sensory nerve activation. This is again in stark contrast to previous findings of the LAR observed in the Brown Norway rat and Guinea-Pig. Future clinical studies, utilising clinically approved long acting muscarinic receptor antagonists, should ascertain whether the mouse model of LAR or other species (i.e. Brown Norway rat and Guinea-Pig models of LAR) are more credible when modeling this feature of asthma.

The final aim of this investigation was to explore the relevance of key allergic effector cells in the LAR by utilising GM mice lacking the relevant immune cell types. The CD4^−/−^ mice displayed a much attenuated response compared with wildtype mice, suggesting a role for CD4^+^ cells in the mechanisms driving the LAR consistent with clinical studies that have identified increased levels of CD4^+^ cells in asthmatic bronchial biopsies compared with healthy controls [34]. Allergen challenge has been associated with less circulating CD4^+^ T-lymphocytes in isolated early responders compared with dual responders (those displaying an EAR and LAR) [35]. Increased levels of CD4^+^ cells have also been found in the BAL fluid of dogs with ragweed allergy at 4 h post allergen challenge [36]. Enhanced levels of CD4^+^ cells in murine models of asthma have also been shown to correlate with the LAR [24]. Depletion and adoptive transfer studies in rats and mice have also deduced a central role for CD4^+^ cells within this response [37–40].

The CD8^−/−^ mice displayed a significantly enhanced response compared to wildtype mice, implicating a protective role for CD8^+^ cells within the LAR. Increased numbers of CD8^+^ T-cells have been found in the airway submucosa of sensitised rats exposed to OVA and depletion of these cells using a monoclonal antibody has been shown to enhance the LAR [40–43]. Adoptive transfer studies have shown that the application of sensitised CD8^+^ cells into rat models of the LAR resulted in suppression of the response upon allergen re-exposure [44]. This LAR suppression has been shown to correlate with enhanced levels of the cytokine IFN-γ in the BAL fluid and the administration of this cytokine to rat models can reduce the LAR [45, 46]. CD8^+^ T-cells are known producers of IFN-γ and so could elicit a protective effect in the allergic airway via this cytokine [47, 48]. Indeed, adoptive transfer studies have shown that application of CD8^+^ γδ T-cells into rat models of allergic asthma resulted in a diminished LAR. Pre-treatment of CD8^+^ γδ T-cells with an antisense oligonucleotide to inhibit IFN-γ before transfer into sensitised recipients resulted in complete recovery of the LAR [49]. It has been shown in numerous investigations that IFN-γ can suppress the proliferation of Th2 type CD4^+^ T-cells and it has been shown to inhibit the infiltration of CD4^+^ cells into the airways in a mouse allergic asthma model [50–54]. Isogai and colleagues showed that transferring CD4^+^ cells into recipient rats resulted in an LAR upon allergen exposure; and in the same study showed the depletion of CD8^+^ cells using monoclonal antibodies resulted in an enhanced LAR [55]. The application of GM mice deficient in CD4^+^and CD8^+^ cell types when exploring the specific mechanisms behind the LAR is novel. On the whole previous studies have employed monoclonal antibodies in allergic asthma models which may target other cell types non-specifically. The results obtained in this investigation correspond with the idea that CD4^+^ T-cells are drivers of the LAR and CD8^+^ T-cells have a protective role in this response which is consistent with the existing literature.

As expected OVA-sensitised wildtype mice had enhanced levels of plasma OVA-specific IgE which also corresponded with the presence of an LAR. The CD4^−/−^mice had significantly decreased levels of plasma OVA-specific IgE and the B-cell deficient mice had negligible levels of plasma allergen specific IgE. The plasma IgE levels were found to be comparable to the corresponding wildtype groups, in the CD8^−/−^ and mast cell deficient mice. OVA sensitised and challenged B-cell^−/−^, Mast cell^−/−^and IgE^−/−^mice displayed a response which was comparable to that seen in wildtype mice, suggesting that these cell types and mediators are not involved in the mechanisms driving the LAR. Interestingly, however, these particular GM mouse strains also exhibited an enhanced baseline response even though they were not sensitised to allergen, making the results difficult to interpret.

Previous pre-clinical studies addressing the role of B-cells, Mast cells and IgE in the LAR have been inconclusive. Mizutani et al. (2012) demonstrated that mice sensitised with OVA-specific IgE displayed an LAR upon OVA challenge [56]. Inhibitors of mast cell products such as cysteinyl leukotrienes have been shown to inhibit the LAR at least partially [13, 57]. Adoptive transfer of CD4^+^ and CD8^+^ cells into a rat model of LAR noted no changes in the EAR and serum IgE levels [40, 46]. Some clinical studies have indicated a prominent role for these cell types and mediators in the LAR. Increased numbers of mast cells and increased levels of IgE in BAL fluid has been associated with the magnitude of the LAR [58, 59]. Patients treated with corticosteroids (which inhibit the LAR) have been shown to have decreased numbers of mast cells in the smooth muscle and epithelium [60]. DSCG, a known mast cell stabiliser, has been shown to inhibit the LAR in asthmatic patients [61–64]. Anti-IgE monoclonal antibodies have also been shown to suppress allergen induced late phase bronchoconstriction [64–67].In contrast, other clinical studies have alluded to mechanisms independent of B-cells, Mast cells and IgE to be important in driving the LAR. In asthmatic patients, Durham et al. (1984) failed to see an association between total or allergen specific IgE and the LAR [68]. Total IgE has been shown to be a poor indicator of the LAR [69]. In the African population, serum levels of IgE have even been reported to be higher in non-asthmatics compared to asthmatics [70]. Studies involving the inhalation of T-cell peptides derived from the feline allergen *Fel d 1* demonstrated the induction of an isolated LAR without an accompanying EAR, implying the LAR to be T-cell dependent and IgE independent [71]. Khan et al. (2000) demonstrated that while Cyclosporin A decreased the LAR in patients, no effects were seen on the EAR indicating that the LAR occurs independently of the B-cell-Mast cell- IgE products axis [72]. Overall, the existing literature on the subject of the involvement of B-cells, Mast cells and IgE in the LAR is sparse and conflicting probably due to the limited tools available for examining the role of these cells and mediators in the LAR. Specificity issues concerning depleting antibodies in asthma investigations have been raised. For example, the antibody MAR-1, targeting the high affinity receptor FcεRI has not only been shown to inhibit mast cell driven processes, but has been shown to activate mast cells [73–75].

Cell deficient strains have been used by others investigating other features of asthma such as AHR and airway inflammation. These investigations have yielded conflicting results and this variation seen in experimental results has been attributed to differences in genetic background, differences in sensitisation and challenge protocols and also the use of adjuvants [76]. A bigger question has also been raised as to the suitability of murine asthma models [77]. It should be noted that there are differences between mice and humans with respect to lung mast cell populations. Humans mast cells are found around the airways and vessels in both the large and small airways and in the parenchyma, whereas in mice, mast cells are mainly located in the trachea and larger airways [78, 79]. In mice, mast cell driven responses such as the EAR are due to the release of 5-HT, compared to human mast cells which secrete histamine and other preformed mediators such as cysteinyl leukotrienes [13–15, 80]. Therefore, it could be that when investigating mast cell responses (and possibly other linked cells and mediators such as B-cells and IgE) the mouse is not the best species to employ.

## Conclusion

In summary, explorations of the murine model of LAR demonstrated that the response was induced by allergen challenge, steroid sensitive and T-cell dependant. These features are consistent with the clinical phenotype observed in clinical allergen challenge studies. However, the requirement for Alum^TM^ adjuvant leads to questions surrounding the clinical validity of the model. The results obtained when exploring a role for airway sensory nerves and the TRPA1 ion channel in this model were not consistent with results obtained in other species such as the Brown Norway rat and Dunkin Hartley guinea-pig. The stark contrast in results should lead to careful consideration of which species to utilise in preclinical asthma models for future studies. Further investigation into the role of airway sensory nerves in clinical investigations is warranted and results from future studies could allow decisions to be made on the optimal species to utilise when modelling the LAR pre-clinically. When exploring the role of key effector cells no conclusions could be made regarding the contribution of mast cells, B-cells and IgE to the LAR. In conclusion, even though certain features of the mouse LAR model are considered to be clinically relevant, other anomalies lead us to question the validity of using a murine pre-clinical model of LAR upon which to assess future asthma therapies.

## Additional file

Additional file 1: The ARRIVE Guidelines Checklist Animal Research: Reporting In Vivo Experiments. (PDF 1068 kb)

## Abbreviations

AHR: Airway hyperresponsiveness
Alum: 20 mg/ml aluminium hydroxide and 20 mg/ml
AUC: Area under the curve
BALF: Bronchoalveolar lavage fluid
BSA: Bovine serum albumin
CD: Cluster of differentiation
ELISA: Enzyme-Linked Immunosorbent Assay
FEV_1_: Forced expiratory volume in 1 s
HRP: Horseradish peroxidase
i.n.: Intranasal
i.p.: Intraperitoneal
i.t.: Intratracheal
IgE: Immunoglobulin E
KO: Knockout
LAR: Late asthmatic response
OVA: Ovalbumin
PBS: Phosphate buffered saline
Penh: Enhanced pause
S.E.M.: Standard error of the mean
TMB: 3,3’,5,5’-Tetramethylbenzidine
WBP: Whole body plethysmograph
WT: Wild type
